# Differential coverage for vaccines in the expanded program on immunization (EPI) among children in rural Pakistan

**DOI:** 10.1016/j.vaccine.2023.03.007

**Published:** 2023-04-17

**Authors:** Shahira Shahid, Sheraz Ahmed, Muhammad Farrukh Qazi, Rafey Ali, Syed Asad Ali, Anita K.M. Zaidi, Najeeha T. Iqbal, Fyezah Jehan, Muhammad Imran Nisar

**Affiliations:** a Department of Pediatrics and Child Health, Aga Khan University, Karachi, Pakistan; b Bill & Melinda Gates Foundation, Seattle, WA, USA

**Keywords:** Vaccine coverage, Vaccine timeliness, Pakistan, Expanded program on immunization, Children, Social determinants

## Abstract

**Background:**

Pakistan has a well-established Expanded Program on Immunization (EPI) however vaccine-preventable diseases still account for high infant and child mortality rates. This study describes the differential vaccine coverage and determinants of vaccine uptake in rural Pakistan.

**Methods:**

From October 2014 to September 2018, we enrolled children younger than 2 years of age from the Matiari Demographic Surveillance System in Sindh, Pakistan. Socio-demographic and vaccination history were collected from all participants. Vaccine coverage rates and timeliness were reported. Socio-demographic variables for missed and untimely vaccination were studied in multivariable logistic regression.

**Results:**

Of the 3140 enrolled children, 48.4 % received all EPI recommended vaccines. Only 21.2 % of these were age appropriate. Around 45.4 % of the children were partially vaccinated, and 6.2 % were unvaccinated. Highest coverage was seen for the first dose of pentavalent (72.8 %), 10-valent Pneumococcal Conjugate Vaccine (PCV10) (70.4 %) and Oral Polio Vaccine (OPV) (69.2 %) and the lowest coverage was for measles (29.3 %) and rotavirus (1.8 %) vaccines. Primary caretakers and wage earners with a higher level of education were protective against missed and untimely vaccination. Enrollment in the 2nd, 3rd and 4th study year was negatively associated with being unvaccinated whereas distance from a major road was positively associated with non-adherence to schedule.

**Conclusion:**

Vaccine coverage was low among children in Matiari, Pakistan, and majority received delayed doses. Parents’ education status and year of study enrollment was protective against vaccine dropout and delayed vaccination whereas geographical distance from a major road was a predictor. Vaccine promotion and outreach efforts may have had a beneficial impact on vaccine coverage and timeliness.

## Highlights

• Vaccine coverage and timeliness were low among children in Matiari, Pakistan.
• 48.4 % of the children were fully vaccinated with all EPI antigens.
• Majority of the doses were delayed, only 21.2 % received all doses on time.
• Parents’ education and study year were protective against missed vaccination.
• Geographical road distance was predictive of untimely vaccination.

## 1 Introduction

Pakistan’s Expanded Program on Immunization (EPI) was established in 1978 and it comprises of vaccination against ten different antigens [1]. It targets approximately 7.5 million children every year across the country and may avert up to 17 % of the childhood mortality [2]. Although improvements to immunization services have been made over the past 40 years [1], Pakistan still reports high under-five mortality rates (65.2 per 1000 live births) and a significant proportion of the deaths are due to vaccine-preventable diseases such as pneumonia, diarrhea, and measles [3]. Vaccine coverage serves as an indicator of the performance of immunization programs and progress towards public health goals. Pakistan has not yet met the WHO’s target of a 95 % vaccination rate. The coverage remains suboptimal in Pakistan with only 66 % of children aged 12–23 months reported to have received all the basic EPI vaccines according to a recent national demographic health survey [4]. The coverage rates for specific vaccines, based on vaccination card and caregiver recall, were reported as 86 % for OPV3, 75 % for Penta3, 64 % for IPV, and 73 % for MCV [4]. There has been a consistent decline in the number of doses administered at later stages in the schedule [5], [6], [7]. This is attributed to limited access to immunisation service centers, particularly in rural areas, lack of vaccinators and an inadequate awareness among caregivers regarding vaccination [8], [9]. The EPI schedule includes five visits in the first year and one in the second year of a child’s life. Previously, little attention has been given to the prevalence of missed opportunities for simultaneous vaccination during a single visit [2]. Additionally, vaccine coverage rates in Pakistan exhibit significant variation between provinces, ranging from 29 % in Balochistan to 80 % in Punjab. Pakistan’s decentralized health system assigns health as a provincial responsibility. There are wide variations within the province for immunization coverage in urban, rural and *peri*-urban areas which largely remain hidden beneath the provincial and national level estimates [4].

We have previously conducted and published a longitudinal study of the impact of the ten-valent pneumococcal conjugate vaccine (PCV10) on nasopharyngeal carriage of vaccine type serotypes in children. The study was performed in two union councils (smallest administrative units) of rural Matiari in the Sindh Province [10], [11]. As a part of the study, we collected data on the uptake of all EPI vaccines among children [10]. Here, we present a secondary analysis of the data to document the differential vaccine drop-out rates and differential vaccine coverage in children less than 2 years of age from 2014 to 18. We also describe sociodemographic characteristics associated with incomplete and delayed vaccination.

## 2 Methods

### 2.1 Study site description

**Fig. 1:**
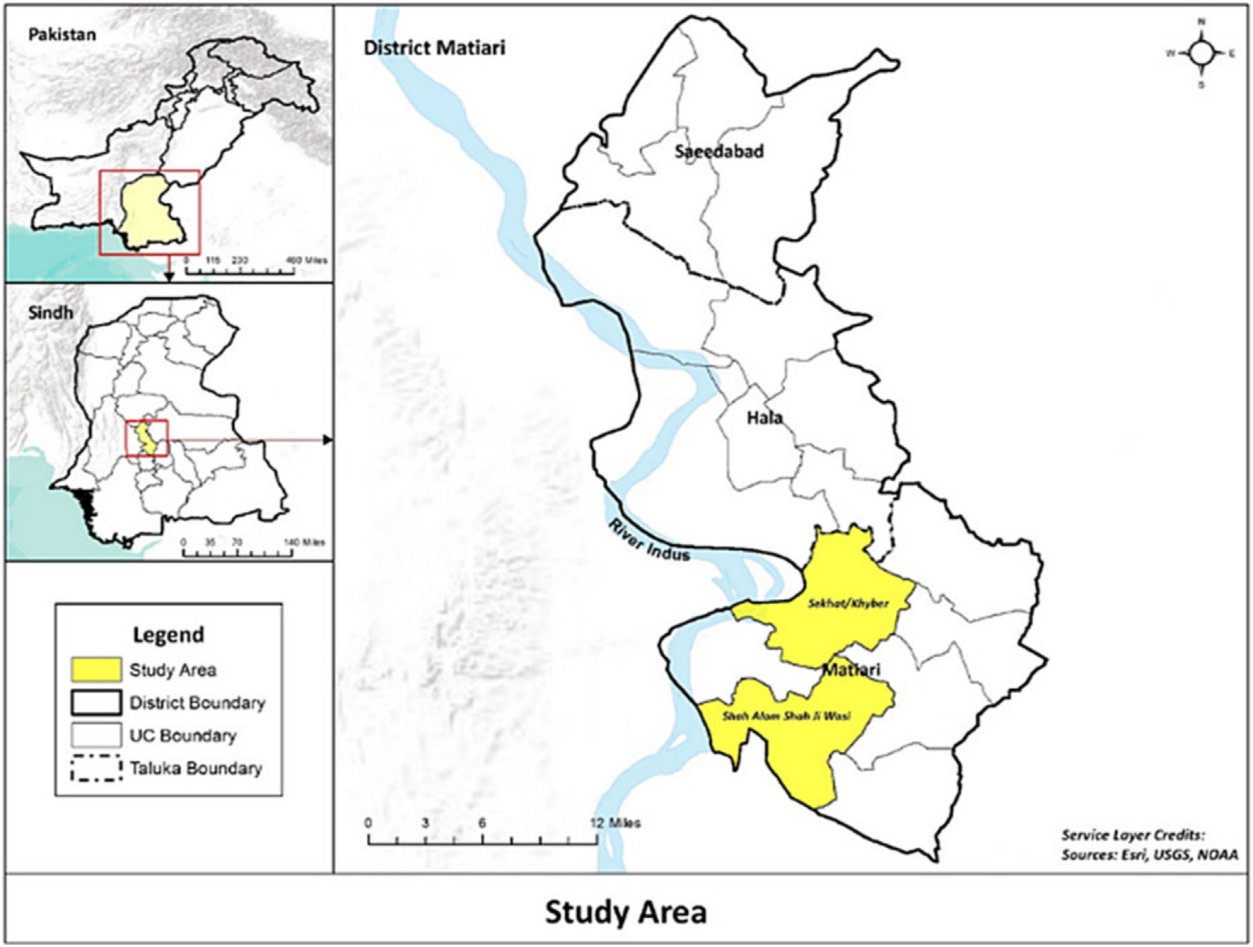
Map of Matiari with highlighted union councils (Khyber and Shah Alam Shah Jee Wasi).

Matiari is a rural district located 182 km from the Aga Khan University in Karachi, Pakistan. We carried out serial cross-sectional surveys from October 2014 to September 2018 in Khyber and Shah Alam Shah Jee Wasi, two Union Councils of Matiari, Sindh (Fig. 1). Trained data collectors visited a total of 262 villages and 11,648 households which covered a population of around 83,000. All participants who provided informed consent were enrolled into a Demographic Surveillance System (DSS). A two-monthly pregnancy and birth surveillance was performed for married women of reproductive age in the catchment area. From 2014 to 2018, yearly surveillance rounds updated information on the 7,889 children aged less than two years in the DSS. Thus, a line listing of all children eligible to receive the EPI vaccines was available. Data on household demographics, recent clinical history including hospitalization and outpatient visits, exposure to household smoke and indoor air pollution was collected by study personnel on smartphones. A brief clinical exam including measurement of fever, respiratory rate, and observation for chest wall indrawing was also performed.

The majority of the population received no formal education and agriculture was their main occupation followed by general labor. The infrastructure was under-developed with poor sanitation and low levels of protected water sources. The DSS community were served by two primary health care centers (PHCs). They provided basic outpatient care and community outreach services, and vaccination services for children and pregnant women in the catchment area. A network of trained community-based health workers called Lady Health Workers (LHWs) performed health promotion activities in the DSS. Each LHW covered approximately 1000 members of the population. Children living in villages without PHCs were visited monthly by governmental vaccination teams. To promote vaccine uptake in the community, we contacted the primary caretakers either on mobile phones or by making a physical visit on the due dates of the vaccination to encourage taking their children for vaccination. Transport was provided where possible. We identified low coverage areas and hired a social mobilization team to sensitize the communities to timely vaccinations.

## 3 EPI schedule

The primary series of immunization included the Bacille Calmette-Guérin (BCG) vaccine at birth, four doses of oral polio vaccine (OPV0) administered at birth, 6, 10 and 14 weeks. Three doses of the pentavalent vaccine which includes the combined diphtheria, tetanus, and pertussis (DPT) antigens, Hepatitis B vaccine (HBV), and *Haemophilus influenzae* type b (HiB) vaccines, at 6, 10 and 14 weeks, three doses of the 10-valent Pneumococcal Conjugate Vaccine (PCV10) at 6, 10 and 14 weeks, two doses of rotavirus vaccine at 6 and 10 weeks of age [1]. This is followed by two doses of measles at 9 and 15 months [12]. Recent changes to the schedule include, the switch from PCV10 to PCV13 vaccine, replacement of the measles vaccine with the measles rubella (MR) vaccine and introduction of the Typhoid Conjugate Vaccine (TCV) to be administered at 9 months.

### 3.1 Statistical analysis

Our study included children who were less than 2 years of age at the time of enrollment. This is the youngest age group likely to have received all the basic EPI antigens. Children’s vaccination status was assessed through either a vaccination card, or maternal recall in cases where a card was not available. The proportion of children who received routine vaccines regardless of the age at which they received them was defined as overall vaccine coverage. Age-appropriate vaccination included those children who received the vaccine within 3 days prior and 28 days after the recommended age as a proportion of the total doses given. Doses received>3 days prior or 28 days after this criterion were considered too early or delayed respectively. Children were classified as fully vaccinated if they received 1 dose of BCG, 4 doses of OPV, 3 doses each of pentavalent and PCV10 by 12 months of age. Measles was most commonly missed vaccine and rotavirus vaccine was introduced in EPI during the final study year, so they were not included in the analysis for being fully vaccinated. Those who missed one or more doses were classified as partially vaccinated and children who had not received any vaccine dose were considered unvaccinated.

For continuous variables, data were expressed as mean ± SD, and for qualitative variables, as frequencies and percentages. A logistic regression model was used to identify sociodemographic determinants for being partially and non-vaccinated as compared to fully vaccinated children, in addition we also described predictors for adherence to the EPI schedule among fully vaccinated children who received all age-appropriate doses. For the purpose of model building all variables with a p-value less than 0.25 in the bivariate analysis were used to build a multivariable model. A backward selection procedure was used to derive a parsimonious model for retaining only variables significant at p-value ≤ 0.05. Multicollinearity among independent variables was assessed using the Variance Inflation Factor (VIF) at a cut-off point of 10. All analysis was performed using STATA version 15.0.

Ethical approval was obtained from Aga Khan University’s Ethical Review Committee (3181-Ped-ERC-14). A written informed consent was obtained from all caretakers before commencing enrollment.

**Data availability**

The data that support the findings of this study are available on request from the corresponding author.

## 4 Results

### 4.1 Sociodemographic characteristics

**Fig. 2:**
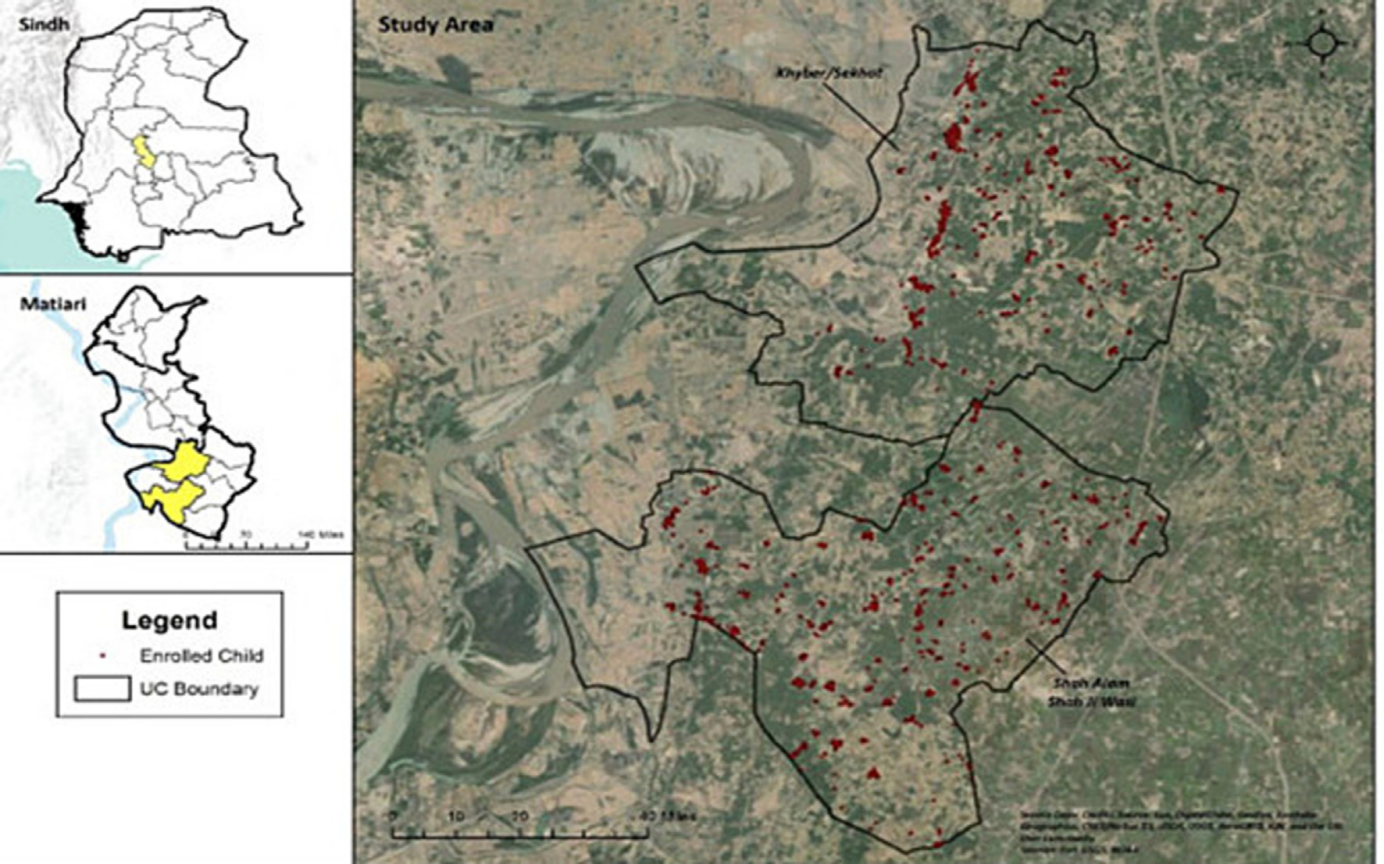
Distribution of the 3140 children enrolled from the DSS.

We approached a total of 4181 households from which 3140 children<2 years were enrolled. Fig. 2 describes the study area along with GIS coordinates mapped for the enrolled children. The flow of study participants is given in supplementary Table S1. Their mean age was 10.5 months, 50.3 % of the enrolled children were male, majority of the primary caretakers, 2596 (82.7 %) and half of the primary wage earners, 1671 (53.2 %) received no formal education. Demographic and clinical characteristics of the study population mostly remained the same across all study years except for age, hospital visits and symptomatic history (Table 1). Age distribution of enrolled children was mostly skewed towards younger age group of 4–11 months except for the year 2014–15 when 61.9 % of the children were aged 12–23 months. The proportion of outpatient visits in the past one month, hospital admissions in the past year and history of symptoms declined over the study period.

**Table 1:** Sociodemographic and clinical characteristics of enrolled children, n = 3140, from 2014 to 2018.

**Year**	**2014/15**	**2015/16**	**2016/17**	**2017/18**
	**N = 771**	**N = 780**	**N = 779**	**N = 810**
**Age, months (Mean ± SD)**	13.3 ± 6.3	9.8 ± 5.8	9.6 ± 5.5	9.4 ± 5.3
0–3 months	55 (7.1 %)	114 (14.6 %)	61 (7.8 %)	75 (9.3 %)
4–11 months	239 (31.0 %)	396 (50.8 %)	479 (61.5 %)	489 (60.4 %)
12–23 months	477 (61.9 %)	270 (34.6 %)	239 (30.7 %)	246 (30.4 %)
**Male**	390 (50.6 %)	381 (48.8 %)	401 (51.5 %)	408 (50.4 %)
**Primary caretaker education**				
no education	637 (82.6 %)	667 (85.5 %)	642 (82.4 %)	650 (80.2 %)
1–5 years	92 (11.9 %)	85 (10.9 %)	83 (10.7 %)	91 (11.2 %)
6 to 10 years	31 (4.0 %)	17 (2.2 %)	38 (4.9 %)	54 (6.7 %)
>10 years	11 (1.4 %)	11 (1.4 %)	16 (2.1 %)	15 (1.9 %)
**Primary wage earner education**				
No education	418 (54.2 %)	438 (56.2 %)	397 (51.0 %)	418 (51.6 %)
1–5 years	194 (25.2 %)	193 (24.7 %)	193 (24.8 %)	181 (22.3 %)
6 to 10 years	92 (11.9 %)	86 (11.0 %)	112 (14.4 %)	129 (15.9 %)
>10 years	67 (8.7 %)	63 (8.1 %)	77 (9.9 %)	82 (10.1 %)
**People in the household, median (IQR)**	8 (6–11)	8 (5–11)	8 (5–11)	8 (6–12)
**Number of rooms in the house, median (IQR)**	1 (1–2)	1 (1–2)	1 (1–2)	1 (1–2)
**Crowding index, Median (IQR)**	6 (4–8)	5.9 (4–7.58)	5 (4–7)	5 (4–7)
**Hospital visits in last month**				
None	287 (37.2 %)	377 (48.3 %)	427 (54.8 %)	520 (64.2 %)
One	194 (25.2 %)	198 (25.4 %)	190 (24.4 %)	215 (26.5 %)
Two or more	290 (37.6 %)	205 (26.3 %)	162 (20.8 %)	75 (9.3 %)
**Hospital Admissions in last year**				
None	733 (95.1 %)	767 (98.3 %)	753 (96.7 %)	793 (97.9 %)
One	36 (4.7 %)	13 (1.7 %)	22 (2.8 %)	12 (1.5 %)
Two or more	2 (0.3 %)	0 (0.0 %)	4 (0.5 %)	5 (0.6 %)
**Smoker in household**	313 (40.6 %)	254 (32.6 %)	263 (33.8 %)	286 (35.3 %)
**Fuel use for cooking**				
Natural Gas	85 (11.0 %)	105 (13.5 %)	146 (18.7 %)	149 (18.4 %)
Wood/paper/straw/crop/animal dung	686 (89.0 %)	675 (86.5 %)	633 (81.3 %)	661 (81.6 %)
**Child exposure to smoke during cooking**	189 (24.5 %)	402 (51.5 %)	423 (54.3 %)	675 (83.3 %)
Symptoms during last two weeks*				
Runny Nose	376 (53.8 %)	469 (60.1 %)	411 (52.8 %)	328 (40.5 %)
Cough	343 (49.1 %)	357 (45.8 %)	311 (39.9 %)	209 (25.8 %)
Fever	401 (57.4 %)	413 (52.9 %)	347 (44.5 %)	302 (37.3 %)
Fast breathing	57 (8.2 %)	9 (1.2 %)	10 (1.3 %)	3 (0.4 %)
Difficulty in breathing	95 (13.6 %)	159 (20.4 %)	181 (23.2 %)	177 (21.9 %)
**Signs**				
Lower chest indrawing	44 (6.3 %)	11 (1.4 %)	12 (1.5 %)	7 (0.9 %)
Tachypnea (as per WHO cutoffs)	97.0 (13.9 %)	46.0 (5.9 %)	53.0 (6.8 %)	17.0(2.1 %)
Hyperthermia	5.0 (0.7 %)	10.0 (1.3 %)	129.0(16.6 %)	43.0 (5.3 %)
Hypothermia	11.0 (1.6 %)	0.0 (0.0 %)	0.0 (0.0 %)	0.0 (0.0 %)

IQR- Interquartile range, ETS- environmental Tobacco Smoke, WHO-World Health Organization *data available for 3068 children.

Tachypnea is defined as: Children younger than 2 months - Greater than or equal to 60 breaths/min, children aged 2–11 months - Greater than or equal to 50 breaths/min, children aged 12–59 month - Greater than or equal to 40 breaths/min. Hypothermia is defined as underarm temperature below 35.0 °C (95.0 °F), Hyperthermia is defined as underarm temperature ≥ 38.0 °C (100.4 °F).

### 4.2 Vaccination coverage and timeliness

**Fig. 3:**
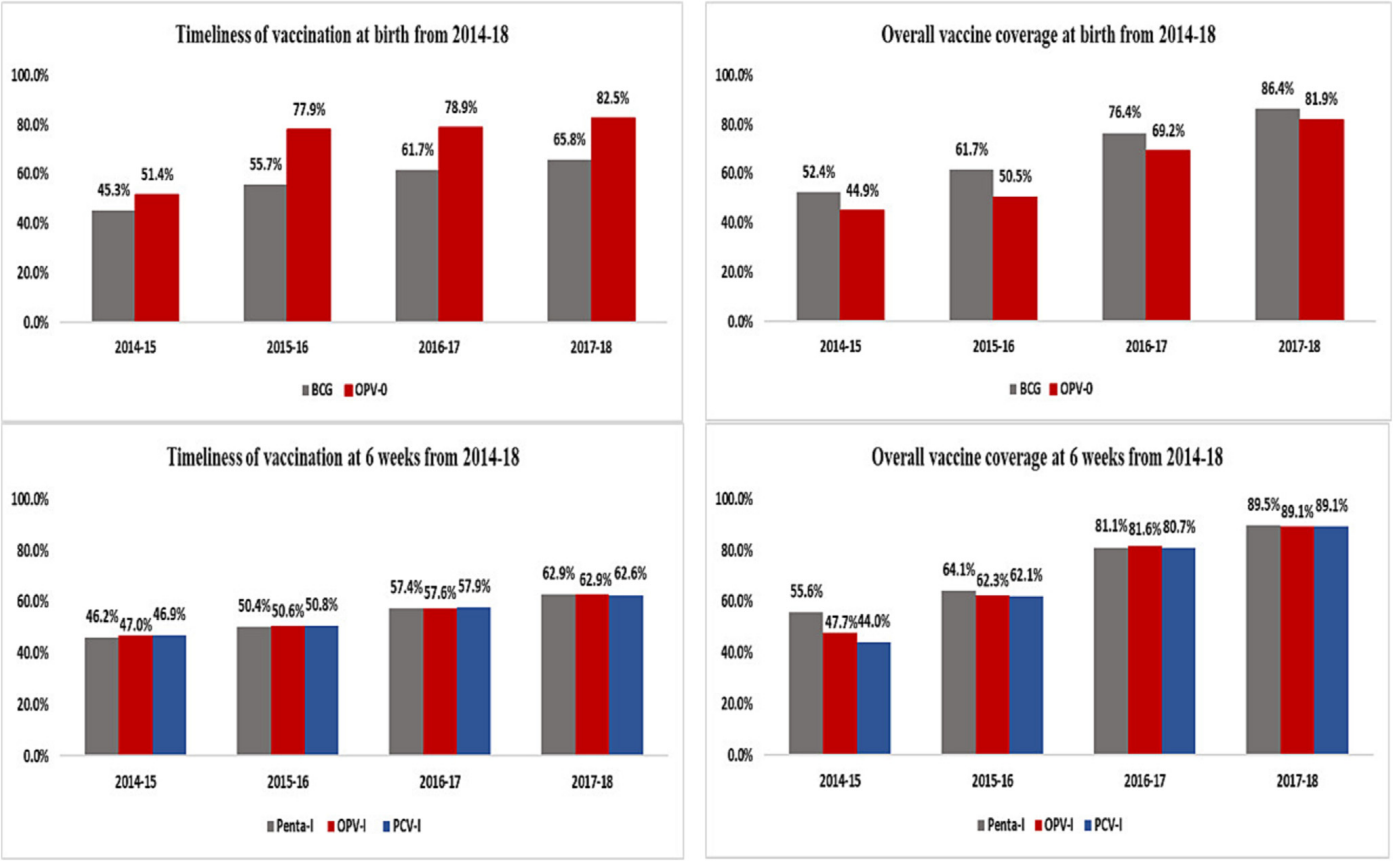

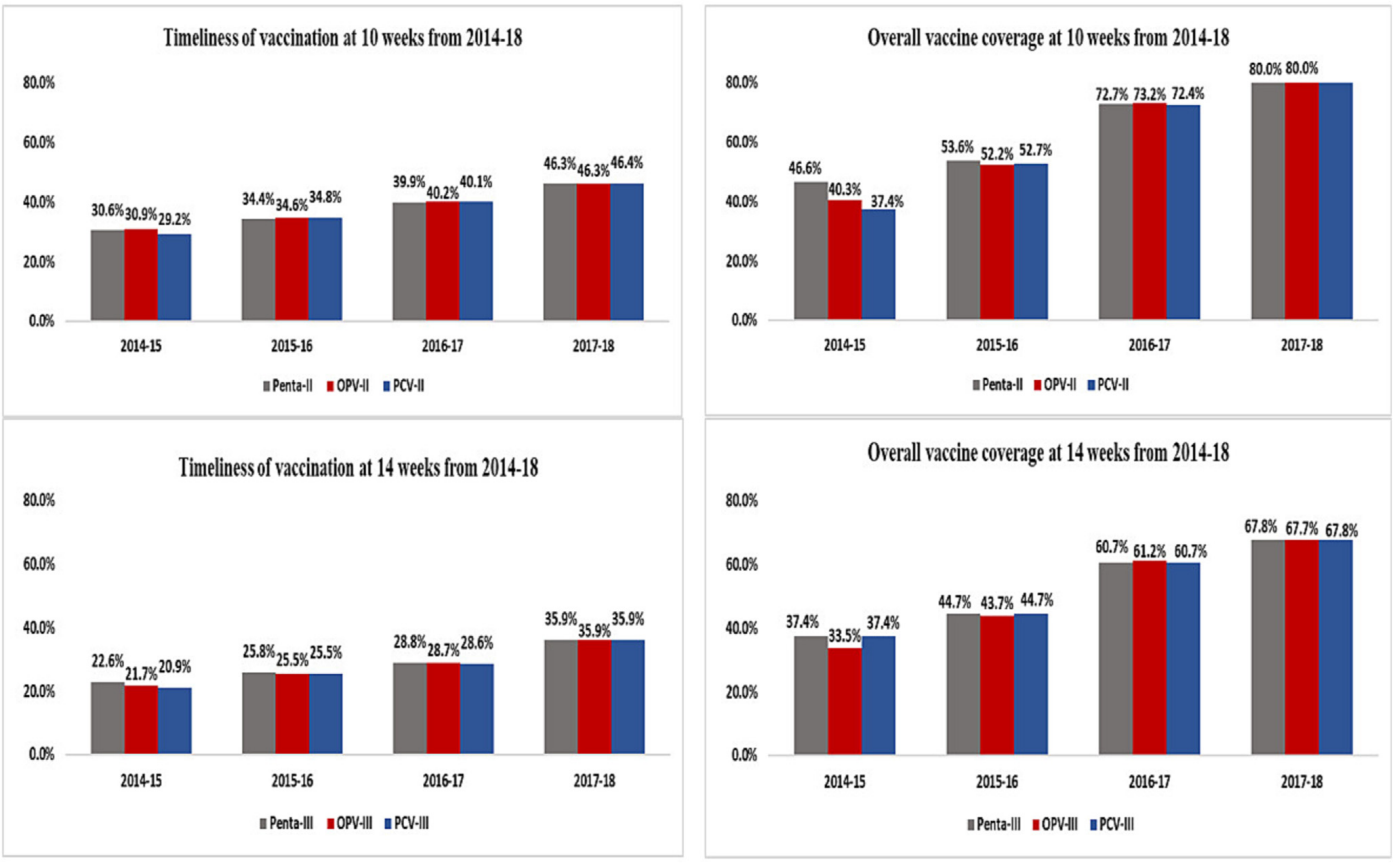
Vaccine coverage and timeliness from 2014 to 2018 for the 3140 enrolled children (a) at birth and 6 weeks (b) at 10 weeks and 14 weeks.

Of the 3140 children enrolled in the study, 48.4 % were fully vaccinated with all EPI antigens except measles and only 9.7 % were fully vaccinated including both doses of the measles vaccine (Table 2). Around 45.4 % children missed one or more antigens and 6.2 % children were completely unvaccinated. Vaccination coverage was the highest for the combination of doses given at 6 weeks of age: penta1 (72.8 %), PCV1 (70.4 %) and OPV1 (69.2 %) and the lowest coverage was seen for measles (29.3 %) followed by rotavirus (1.8 %) vaccines (Table 2). For multi-dose vaccines including OPV, PCV10, pentavalent, rotavirus and measles, the proportion of vaccinated children progressively decreased with each successive dose; it decreased from 61.8 % for OPV0 to 51.7 % for OPV3 and from 72.8 % for penta1 to 53.8 % for penta3 (Fig. 3 a and b).

**Table 2:** Coverage for EPI recommended vaccines among children <2 years of age.

**EPI Schedule**	**Antigen-Dose number**	**Not received** **n( %)**	**Received** **n( %)**
		**N = 3140**	
**At Birth**	BCG	960 (30.6 %)	2180 (69.4 %)
	OPV-0	1198 (38.2 %)	1942 (61.8 %)
**6 Weeks**	OPV-I	928 (29.6 %)	2212 (70.4 %)
	PCV-I	966 (30.8 %)	2174 (69.2 %)
	Penta-I	854 (27.2 %)	2286 (72.8 %)
	Rota-I	3072 (97.8 %)	68 (2.2 %)
**10 Weeks**	OPV-II	1204 (38.3 %)	1936 (61.6 %)
	PCV-II	1228 (39.1 %)	1912 (60.9 %)
	Penta-II	1149 (36.6 %)	1991 (63.4 %)
	Rota-II	3072 (97.8 %)	56 (1.8 %)
**14 Weeks**	OPV-III	1516 (48.3 %)	1624 (51.7 %)
	PCV-III	1481 (47.2 %)	1659 (52.8 %)
	Penta-III	1481 (41.2 %)	1659 (53.8 %)
	IPV-I	2760 (87.9 %)	380 (12.1 %)
**9 Months**	Measles-I	2218 (70.7 %)	922 (29.3 %)
**15 Months**	Measles-II	2814 (89.6 %)	326 (10.4 %)

A differential in coverage was observed between antigens given at the same visit; BCG (69.4 %) had a greater coverage than OPV0 (61.8 %). The first dose coverage of pentavalent vaccine was higher than PCV and OPV during the first two years of the study. This difference in coverage was overcome by the final study year for the same antigens given at 10 weeks and 14 weeks.

Among the 1522 children who were fully vaccinated, only 21.2 % received all doses on time. Out of the total vaccine doses given, few were given early, and majority of the doses were delayed (Table 3.). There was a significant decline in timeliness for later doses of multi-dose vaccines, around 56 % children received the first dose of pentavalent, PCV and OPV on time, but only 39 % and 29.4 % received the second and third doses as per schedule respectively. Age-appropriate coverage for vaccines given at birth, were 75 % for OPV0 and 58.7 % for BCG. Vaccine coverage and timeliness improved for all vaccines and doses over the study period (Fig. 3a and b).

**Table 3:** Adherence to EPI schedule among vaccinated children <2 years of age.

**EPI Schedule**	**Antigen-Dose number**		**Age-appropriate**	**Delayed**	**Early**
		**N**	**n( %)**	**n( %)**	**n( %)**
**At Birth**	BCG	2180	1279(58.7 %)	901(41.3 %)	–
	OPV-0	1942	1457(75 %)	485(25 %)	–
**6 Weeks**	OPV-I	2212	1239(56 %)	928(42 %)	45(2 %)
	PCV-I	2174	1221(56.2 %)	910(41.9 %)	43(2 %)
	Penta-I	2286	1269(55.5 %)	969(42.4 %)	48(2.1 %)
	Rota-I	68	35(51.5 %)	33(48.5 %)	0 (0 %)
**10 Weeks**	OPV-II	1936	766(39.6 %)	1147(59.3 %)	23(1.2 %)
	PCV-II	1912	754(39.4 %)	1133(59.3 %)	25(1.3 %)
	Penta-II	1991	780(39.2 %)	1185(59.5 %)	26 (1.3 %)
	Rota-II	56	20(35.7 %)	36(64.3 %)	–
**14 Weeks**	OPV-III	1624	477(29.4 %)	1138(70.1 %)	9(0.6 %)
	PCV-III	1598	469(29.4 %)	1120(70.1 %)	10(0.6 %)
	Penta-III	1659	488(29.4 %)	1161(70 %)	10(0.6 %)
	IPV-I	380	141(37.1 %)	238(62.6 %)	1 (0.3 %)
**9 Months**	Measles-I	922	345(37.4 %)	422(45.8 %)	155(16.8 %)
**15 Months**	Measles-II	326	134(41.1 %)	119(36.5 %)	73(22.4 %)

### 4.3 Determinants for complete and timely vaccination

Table 4 describes the bivariate and multivariate analysis for the predictors of being partially/unvaccinated. The final model showed a positive association with children living in households where wood/paper/straw/crop/animal dung were used as a cooking fuel (aOR 1.8 95 %CI 1.4–2.2) and a negative association was seen in children 2.5 younger than fully vaccinated children (aOR 0.9 95 %CI 0.9–1.0), primary caretaker’s education of 6–10 years (aOR 0.6 95 %CI 0.4–0.9), >10 years (aOR 0.4 95 %CI 0.2–0.8), primary wage earner’s education of 1–5 years (aOR 0.8 95 %CI 0.6–0.9), 6–10 years (aOR 0.7 95 %CI 0.5–0.9) and > 10 years (aOR 0.5 95 %CI 0.4–0.7) and being enrolled in the second (aOR 0.5 95 %CI 0.4–0.7), third (aOR 0.3 95 %CI 0.2–0.4), and fourth (aOR 0.2 95 %CI 0.2–0.3) study year.

**Table 4:** Factors associated with fully vaccinated status (n = 3140).

**Characteristics**	**Partially/ Unvaccinated**	**Fully vaccinated**	**OR (95 %CI)**	**aOR(95 %CI)**	**p-value**
	**N = 1,618**	**N = 1,522**			
**Age, months (Mean ± SD)**	9.3 ± 6.2	11.8 ± 5.4	0.9(0.9,0.9)	0.9(0.9,0.9)	<0.001
**Gender**					
Male	795 (50.3 %)	785 (49.7 %)	Ref		
Female	823 (52.8 %)	737 (47.2 %)	1.1(1.0,1.3)		
**Wasting (Weight for Height), n = 813**					
Normal	264 (38.9 %)	414 (61.1 %)	Ref		
Wasted	61 (45.2 %)	74 (54.8 %)	1.3(0.9,1.9)		
**Stunting (Height for Age), n = 803**					
Normal	207 (40.9 %)	299 (59.1 %)	Ref		
Stunted	114 (38.4 %)	183 (61.6 %)	0.9(0.7,1.2)		
**Under-weight (Weight for Age), n = 808**					
Normal	198 (38.4 %)	318 (61.6 %)	Ref		
Under-weight	122 (41.8 %)	170 (58.2 %)	1.2(0.9,1.5)		
**Primary care taker's education**					
no education	1,406 (54.2 %)	1,190 (45.8 %)	Ref	Ref	
1–5 years	154 (43.9 %)	197 (56.1 %)	0.7(0.5,0.8)	0.8(0.6,1.1)	0.125
6 to 10 years	44 (31.4 %)	96 (68.6 %)	0.4(0.3,0.6)	0.6(0.4,0.9)	0.025
>10 years	14 (26.4 %)	39 (73.6 %)	0.3(0.2,0.6)	0.4(0.2,0.8)	0.014
**Primary wage earner's education**					
no education	953 (57.0 %)	718 (43.0 %)	Ref	Ref	
1–5 years	385 (50.6 %)	376 (49.4 %)	0.8(0.6,0.9)	0.8(0.6,0.9)	0.011
6 to 10 years	180 (43.0 %)	239 (57.0 %)	0.6(0.5,0.7)	0.7(0.5,0.9)	0.003
>10 years	100 (34.6 %)	189 (65.4 %)	0.4(0.3,0.5)	0.5(0.4,0.7)	<0.001
**Road distance to health facility, n = 3,139**					
<2 Km	80 (48.8 %)	84 (51.2 %)	Ref		
2–5 Km	578 (47.7 %)	633 (52.3 %)	1.0(0.7,1.3)		
>5 Km	960 (54.4 %)	804 (45.6 %)	1.3(0.9,1.7)		
**Children residing within 2 km buffer from a major road, n = 3,139**					
Yes	1,304 (51.4 %)	1,235 (48.6 %)	1.0(0.8,1.1)		
No	314 (52.3 %)	286 (47.7 %)	Ref		
**Crowding index, Median(IQR)**	5.5 (4.0–7.0)	5.5 (4.0–7.0)	1.0(1.0,1.0)		
**Hospital visits in the last month**					
None	795 (49.3 %)	816 (50.7 %)	Ref		
One	409 (51.3 %)	388 (48.7 %)	1.1(0.9,1.3)		
Two or more	414 (56.6 %)	318 (43.4 %)	1.3(1.1,1.6)		
**Hospital Admissions in the last year**	59 (62.8 %)	35 (37.2 %)	1.6(1.1,2.5)		
**Smoker in household**	591 (53.0 %)	525 (47.0 %)	1.1(0.9,1.3)		
**Fuel used for cooking**					
Natural Gas	162 (33.4 %)	323 (66.6 %)	Ref	Ref	
Others	1,456 (54.8 %)	1,199 (45.2 %)	2.4(2.0,3.0)	1.8(1.4,2.2)	<0.001
**Child exposure to smoke during cooking**	797 (47.2 %)	892 (52.8 %)	0.7(0.6,0.8)		
**Symptoms during last two weeks, n = 3,068**					
Runny Nose	813 (51.3 %)	771 (48.7 %)	1(0.9,1.1)		
Cough	656 (53.8 %)	564 (46.2 %)	1.2(1,1.3)		
Fever	798 (54.5 %)	665 (45.5 %)	1.3(1.1,1.5)		
Fast breathing	48 (60.8 %)	31 (39.2 %)	1.5(0.9,2.3)		
Difficulty in breathing	336 (54.9 %)	276 (45.1 %)	1.2(1.0,1.4)		
**Signs, n = 3,068**					
Hypothermia	4 (36.4 %)	7 (63.6 %)	Ref		
Hyperthermia	96 (51.3 %)	91 (48.7 %)	1.8(0.5,6.5)		
Tachypnea (as per WHO cutoffs)	118 (55.4 %)	95 (44.6 %)	1.2(0.9,1.6)		
Lower chest indrawing	47 (63.5 %)	27 (36.5 %)	1.7(1.0,2.7)		
**Year of enrollment**					
2014/15	491 (63.7 %)	280 (36.3 %)	Ref	Ref	
2015/16	456 (58.5 %)	324 (41.5 %)	0.8(0.7,1)	0.5(0.4,0.7)	<0.001
2016/17	348 (44.7 %)	431 (55.3 %)	0.5(0.4,0.6)	0.3(0.2,0.4)	<0.001
2017/18	323 (39.9 %)	487 (60.1 %)	0.4(0.3,0.5)	0.2(0.2,0.3)	<0.001
**Temperature ˚C, (Mean ± SD)**	32.2 ± 4.8	32.4 ± 4.7	1.0(1.0,1.0)		

IQR- Interquartile range, ETS- environmental Tobacco Smoke, WHO-World Health Organization.

Tachypnea is defined as: Children younger than 2 months - Greater than or equal to 60 breaths/min, children aged 2–11 months - Greater than or equal to 50 breaths/min, children aged 12–59 month - Greater than or equal to 40 breaths/min. Hypothermia is defined as underarm temperature below 35.0 °C (95.0 °F), Hyperthermia is defined as underarm temperature ≥ 38.0 °C (100.4 °F).

Table 5 describes the predictors for non-adherence to schedule, it was positively associated with households where wood/paper/straw/crop/animal dung were used as a cooking fuel (aOR 1.9 95 %CI 1.4–2.6), and negatively associated with households located within 2 km of a major road (aOR 0.4 95 %CI 0.3–0.6), primary wage earner’s education status of 6–10 years (aOR 0.6 95 %CI 0.4–0.9), and > 10 years (aOR 0.6 95 %CI 0.4–0.9).

**Table 5:** Factors associated with age-appropriate vaccination (n = 3140).

**Characteristics**	**Untimely vaccination**	**All vaccines on time**	**OR (95 %CI)**	**aOR(CI)**	**p-value**
	**N = 1,199**	**N = 323**			
**Age, months (Mean ± SD)**	12.34 ± 5.30	9.68 ± 5.05	1.1(1.1,1.1)	1.1(1.1,1.1)	<0.001
**Gender**					
Male	614 (78.22 %)	171 (21.78 %)	Ref		
Female	585 (79.38 %)	152 (20.62 %)	1.1(0.8,1.4)		
**Wasting (Weight for Height), n = 488**					
Normal	296 (71.50 %)	118 (28.50 %)	Ref		
Wasted	56 (75.68 %)	18 (24.32 %)	1.2(0.7,2.2)		
**Stunting (Height for Age), n = 482**					
Normal	208 (69.57 %)	91 (30.43 %)	Ref		
Stunted	141 (77.05 %)	42 (22.95 %)	1.5(1,2.2)		
**Under-weight (Weight for Age), n = 488**					
Normal	220 (69.18 %)	98 (30.82 %)	Ref		
Under-weight	133 (78.24 %)	37 (21.76 %)	1.6(1,2.5)		
**Primary care taker's education status**					
No education	959 (80.59 %)	231 (19.41 %)	Ref		
1–5 years	150 (76.14 %)	47 (23.86 %)	0.8(0.5,1.1)		
6 to 10 years	68 (70.83 %)	28 (29.17 %)	0.6(0.4,0.9)		
>10 years	22 (56.41 %)	17 (43.59 %)	0.3(0.2,0.6)		
**Primary wage earner's education status**					
No education	590 (82.17 %)	128 (17.83 %)	Ref	Ref	
1–5 years	305 (81.12 %)	71 (18.88 %)	0.9(0.7,1.3)	1.0(0.7,1.4)	0.807
6 to 10 years	170 (71.13 %)	69 (28.87 %)	0.5(0.4,0.8)	0.6(0.4,0.9)	0.006
>10 years	134 (70.90 %)	55 (29.10 %)	0.5(0.4,0.8)	0.6(0.4,0.9)	0.017
**Road distance to health facility, n = 1,521**					
<2 Km	62 (73.81 %)	22 (26.19 %)	Ref		
2–5 Km	464 (73.30 %)	169 (26.70 %)	1(0.6,1.6)		
>5 Km	672 (83.58 %)	132 (16.42 %)	1.8(1.1,3)		
**Children residing within 2 km buffer from a major road, n = 1,521**					
Yes	945 (76.52 %)	290 (23.48 %)	0.4(0.3,0.6)	0.4(0.3,0.6)	<0.001
No	253 (88.46 %)	33 (11.54 %)	Ref	Ref	
**Crowding index, Median(IQR)**	5.50 (4.0–7.5)	5.0 (4.0–7.0)	1.1(1,1.1)		
**Hospital visits in last month**					
None	652 (79.90 %)	164 (20.10 %)	Ref		
One	294 (75.77 %)	94 (24.23 %)	0.8(0.6,1)		
Two or more	253 (79.56 %)	65 (20.44 %)	1(0.7,1.4)		
**Hospital Admissions in the last year**	29 (82.86 %)	6 (17.14 %)	1.3(0.5,3.2)		
**Smoker in household**	418 (79.62 %)	107 (20.38 %)	1.1(0.8,1.4)		
**Fuel used for cooking**					
Natural Gas	217 (67.18 %)	106 (32.82 %)	Ref	Ref	
Others	982 (81.90 %)	217 (18.10 %)	2.2(1.7,2.9)	1.9(1.4,2.6)	<0.001
**Child exposure to smoke during cooking**	732 (82.06 %)	160 (17.94 %)	1.6(1.2,2)		
**Symptoms during the last two weeks, n = 1,487**					
Runny Nose	601 (77.95 %)	170 (22.05 %)	0.9(0.7,1.2)		
Cough	440 (78.01 %)	124 (21.99 %)	0.9(0.7,1.2)		
Fever	529 (79.55 %)	136 (20.45 %)	1.1(0.9,1.4)		
Fast breathing	28 (90.32 %)	3 (9.68 %)	2.6(0.8,8.5)		
Difficulty in breathing	203 (73.55 %)	73 (26.45 %)	0.7(0.5,0.9)		
**Signs, n = 1,487**					
Lower chest indrawing	25 (92.59 %)	2 (7.41 %)	3.4(0.8,14.6)		
Tachypnea (as per WHO cutoffs)	83 (87.37 %)	12 (12.63 %)	1.9(1,3.6)		
Hypothermia	7 (100.0 %)	0 (0.00 %)	Ref		
Hyperthermia	69 (75.82 %)	22 (24.18 %)	0.8(0.5,1.4)		
**Year of enrollment**					
2014/15	250 (89.29 %)	30 (10.71 %)	Ref		
2015/16	267 (82.41 %)	57 (17.59 %)	0.6(0.3,0.9)		
2016/17	330 (76.57 %)	101 (23.43 %)	0.4(0.3,0.6)		
2017/18	352 (72.28 %)	135 (27.72 %)	0.3(0.2,0.5)		
**Temperature ˚C, (Mean ± SD)**	32.36 ± 4.74)	32.61 ± 4.63)	1(1,1)		

IQR- Interquartile range, ETS- environmental Tobacco Smoke, WHO-World Health Organization.

Tachypnea is defined as: Children younger than 2 months - Greater than or equal to 60 breaths/min, children aged 2–11 months - Greater than or equal to 50 breaths/min, children aged 12–59 month - Greater than or equal to 40 breaths/min. Hypothermia is defined as underarm temperature below 35.0 °C (95.0 °F), Hyperthermia is defined as underarm temperature ≥ 38.0 °C (100.4 °F).

## 5 Discussion

Our study found that one out of every 10 children less than two years of age in Matiari, Pakistan were completely unvaccinated. Almost 45.4 % of children failed to complete their vaccination schedule. Among those who completed their schedule through routine or outreach activities, only 20 % received all doses on time. Despite this, both coverage and timeliness improved for all doses during the study period between 2014 and 2018. Vaccination coverage was the highest for the first dose of the pentavalent/PCV/OPV combination and the most commonly missed dose was that of measles.

The coverage rates in our study are lower than previous estimates from the same union councils [13]. Moreover, our reported data indicates a discrepancy with national estimates. The coverage rate in Sindh province was 48.8 %. The urban areas of Sindh had a higher coverage rate of 62.9 % compared to the rural areas where the coverage rate was 36.8 % [4]. It is important to note that these estimates consider the measles vaccine, when we included measles in our analyses the vaccination coverage dropped to 9.2 %. In our study, the coverage for most antigens remained at 50–70 %, which is contrary to the PDHS estimates of > 70 % coverage for most vaccines in the Sindh province [4]. One explanation for this could be that these national and provincial level data ignore the disparities within the smaller administrative units such as districts and union councils. It is important to recognize this difference as it will help us in reaching those children who are in highest need of vaccines.

The reasons for gaps in vaccine coverage in Matiari are multifaceted. Matiari is a rural district in Sindh, which faces several challenges such as chronic poverty, underdeveloped infrastructure, illiteracy and limited healthcare facilities to meet the needs of the growing population. In a previously published report, one out of every-three immunization centers lacked critical components for vaccination such as the presence of a cold box/refrigerator, vaccinator, and vaccination equipment [14]. Consequently, children are left vulnerable to outbreaks of vaccine preventable diseases. From 2014 to 2018, healthcare facilities in Matiari overseen by a local NGO reported an increase in diarrhea cases from 24,505 to 30,479 among children under 5 years of age, while the reported cases of pneumonia rose from 2,340 to 8,148 during the same period [15]. Thus, to improve coverage in Matiari, interventions should address both supply-side and demand-side issues such as a weak health-system, lack of accurate surveillance data, global pressure for polio eradication, security risks for community-based activities, and misconceptions regarding vaccines among the community [16].

We revealed key socioeconomic and demographic characteristics which remain central challenges to the achievement of childhood immunization. Consistent with prior literature on vaccination, we found primary caregiver and wage earner’s education level to be protective against missed and delayed vaccination [17], [18], [19]. A previous study from Sindh by Jamal et al. revealed that female caregivers prioritized their child's health and made decisions on vaccination independently without involvement of male household members [20]. However, unawareness was a bigger issue than refusal among caregivers as majority had limited knowledge regarding vaccination [21], [22]. Thus, the root cause of low coverage rates is related to upstream determinants such as education. Based on our census data, 56.9 % of the women in Matiari received no education, and only 0.8 % attended school till secondary level. To promote better retention rates among female students, it is essential to promote awareness through social mobilization, enhance the infrastructure and provide basic amenities, such as water and sanitation facilities, as well as transportation services for girls in rural areas. In our study, majority (96 %) of the households were located>2 kms from a health facility and most individuals travelled on foot or by rickshaw, making travel with young children difficult, and time-consuming. Consistent with previous literature, households located>2 kms away from a main road were significantly associated with untimely vaccination [23]. Thus, children in these hard-to-reach areas can benefit from enhanced mobile outreach strategy and supplementary immunisation activities [24]. Previously, the use of mobile phone text message vaccination reminders, and small mobile conditional cash transfers have shown to be effective in improving vaccination in Pakistan [25], [26]. Among household-specific predictors, the use of wood/paper/straw/crop/animal dung being for cooking had higher odds of children being unvaccinated. The use of inexpensive cooking fuels can be considered an indirect indicator of the socioeconomic status here [27].

Khowaja et al. previously reported a lower presence of outreach vaccination support by LHWs (34–43 %) in the DSS as compared to other union councils in Matiari [13]. The association of year of enrollment with vaccine completeness reflects an increase in vaccine promotion and enhanced outreach activities by the study staff. Coverage levels were, however, far below the desired levels to achieve eradication of poliomyelitis and elimination of measles. In 2017, Pakistan reported the third-highest number of children under one year of age who missed out on the first dose (1.2 million), a possible reason could be that measles is administered at 9 months which is much later in the schedule [28]. Attrition was high for the third dose of the pentavalent/OPV/PCV combination given at 14 weeks of age. Although BCG and OPV0 are co-administered at birth, 16.3 % of the children missed the opportunity to be simultaneously vaccinated with BCG. This is consistent with a national survey, which found that 10.9 % of the children experienced MOSVs for BCG. According to Hussain et al. the reason for delayed BCG vaccination compared to OPV0 in Pakistan may be due to the practice of opening 20-dose BCG vials only when enough children were present [29]. The late administration of BCG has been associated with higher mortality previously [30].

During each visit, we noted that the drop-out rates for PCV were higher than those for the pentavalent vaccine, which is in line with findings from a national survey [29]. Caregivers in Sindh preferred fewer vaccines being co-administered to their child to avoid adverse events and discomfort [20], [29]. Thus, they opted to receive the combination vaccine (pentavalent vaccine) which protects against five diseases as opposed to PCV. This is in contrast with high-income countries where the use of combination vaccines served as a positive externality by improving the coverage and timeliness of other antigens administered during the same visit [31], [32]. Thus, efforts promoting co-administration should address safety concerns among both parents and healthcare professionals.

We studied a large cohort of children in a rural setting which can give a good insight into the present situation. Our population was well defined, and our sample size appropriately calculated for a bivariate logistic regression analysis. Our study had some limitations. In cases where vaccination cards were not available, maternal recall was used which can lead to recall bias. Vaccination cards were available for fewer children in our study which is consistent with previous literature from Pakistan in which EPI card retention ranged from 24 to 33 % [33]. The exclusion of children who did not have vaccination card from the analysis, would have substantially reduced our sample size. Vaccination coverage and timeliness among children could be influenced by many other factors, including, knowledge, attitudes, and practices of the parents and providers. Rotavirus was introduced in the later study years and its coverage was not included in analyses.

## 6 Conclusion

Vaccine coverage among children<2 years of age in Matiari, Pakistan was lower than other national and provincial level estimates, and only 20 % of the children received their doses on time. It is important to identify and delineate clusters of population where vaccine uptake is low and evaluate the reasons for them getting missed by health systems. This approach can be used by stakeholders to implement targeted interventions at district and union council level to ensure complete vaccination in children from households in geographically distant areas, households in the lowest wealth quintile and children of mothers with no education.

## Funding

This work was supported by the Bill & Melinda Gates Foundation through grant ID #OPP1111303. Funder had no role in collection, analysis, or interpretation of the data.

## CRediT authorship contribution statement

**Shahira Shahid:** Writing – original draft, Writing – review & editing, Formal analysis, Visualization. **Sheraz Ahmed:** Writing – review & editing, Supervision, Project administration. **Muhammad Farrukh Qazi:** Writing – review & editing, Data curation, Software, Formal analysis, Visualization. **Rafey Ali:** Writing – review & editing, Software, Formal analysis, Visualization. **Asad Ali:** Writing – review & editing, Conceptualization, Methodology, Funding acquisition, Supervision. **Anita K.M. Zaidi:** Writing – review & editing, Supervision. **Najeeha T. Iqbal:** Writing – review & editing, Methodology, Formal analysis, Supervision. **Fyezah Jehan:** Conceptualization, Methodology, Writing – review & editing, Funding acquisition. **Muhammad Imran Nisar:** Conceptualization, Methodology, Formal analysis, Writing – original draft, Writing – review & editing, Visualization, Supervision, Project administration, Funding acquisition.

## Declaration of Competing Interest

The authors declare that they have no known competing financial interests or personal relationships that could have appeared to influence the work reported in this paper.

## Data Availability

Data will be made available on request.

## References

[1] Hasan Q., Bosan A.H., Bile K.M. (2010). A review of EPI progress in Pakistan towards achieving coverage targets: Present situation and the way forward. East Mediterr Health J.

[2] Pakistan Go. Fact sheet of EPI 2022 [Available from: http://www.epi.gov.pk/wp-content/uploads/2021/08/Fact-File-of-EPI-Pakistan.pdf.

[3] Dunne E.M., Satzke C., Ratu F.T., Neal E.F.G., Boelsen L.K., Matanitobua S. (2018). Effect of ten-valent pneumococcal conjugate vaccine introduction on pneumococcal carriage in Fiji: Results from four annual cross-sectional carriage surveys. Lancet Glob Health.

[4] ICF NIoPSNPa. 2017-18 Pakistan Demographic and Health Survey Key Findings. Islamabad, Pakistan, and Rockville, Maryland, USA: NIPS and ICF. 2019.

[5] Ahmed T.A.S., Aliaga A., Arnold F., Ayub M., Bhatti M. (1992, 1992,).

[6] NIPS (2012). Pakistan Demographic and Health Survey 2012–13. Secondary Pakistan Demographic and Health Survey.

[7] Pakistan Demographic and Health Survey 2006-07 Islamabad. Pakistan: National Institute of Population Studies and Macro International Inc. 2008.

[8] Owais A., Khowaja A.R., Ali S.A., Zaidi A.K. (2013). Pakistan's expanded programme on immunization: An overview in the context of polio eradication and strategies for improving coverage. Vaccine.

[9] Bugvi A.S., Rahat R., Zakar R., Zakar M.Z., Fischer F., Nasrullah M. (2014). Factors associated with non-utilization of child immunization in Pakistan: evidence from the demographic and health survey 2006–07. BMC Public Health.

[10] Nisar M.I., Ahmed S., Jehan F., Shahid S., Shakoor S., Kabir F. (2021). Direct and indirect effect of 10 valent pneumococcal vaccine on nasopharyngeal carriage in children under 2 years of age in Matiari. Pakistan Vaccine.

[11] Nisar M.I., Jehan F., Shahid S., Shakoor S., Kabir F., Hotwani A. (2021). Methods for estimating the direct and indirect effect of 10 valent pneumococcal vaccine on nasopharyngeal carriage in children under 2 years in Matiari. Pakistan MethodsX.

[12] Aslam F., Yue Y., Aziz M. (2021). Introduction of typhoid vaccine in the expanded immunization program of Pakistan. Hum Vaccin Immunother.

[13] Khowaja A.R., Zaman U., Feroze A., Rizvi A., Zaidi A.K.M. (2015). Routine EPI Coverage: Subdistrict inequalities and reasons for immunization failure in a rural setting in Pakistan. Asia Pacific J Public Health.

[14] Siddiqi D.A., Abdullah S., Dharma V.K., Khamisani T., Shah M.T., Setayesh H. (2022). Assessment of vaccination service delivery and quality: A cross-sectional survey of over 1300 health facilities from 29 districts in Sindh, Pakistan conducted between 2017–18. BMC Health Serv Res.

[15] Sindh P. Matiari District Health Facilities 2023 [Available from: https://pphisindh.org/home/district-info.php?id=17].

[16] Haq Z., Shaikh B.T., Tran N., Hafeez A., Ghaffar A. (2019). System within systems: Challenges and opportunities for the expanded programme on immunisation in Pakistan. Health Res Policy Syst.

[17] Priya P.K., Pathak V.K., Giri A.K. (2020). Vaccination coverage and vaccine hesitancy among vulnerable population of India. Hum Vaccin Immunother.

[18] Asif A.M., Akbar M., Tahir M.R., Arshad I.A. (2019). Role of maternal education and vaccination coverage: Evidence from Pakistan demographic and health survey. Asia Pac J Public Health.

[19] Forshaw J., Gerver S.M., Gill M., Cooper E., Manikam L., Ward H. (2017). The global effect of maternal education on complete childhood vaccination: A systematic review and meta-analysis. BMC Infect Dis.

[20] Jamal D., Zaidi S., Husain S., Orr D.W., Riaz A., Farrukhi A.A. (2020). Low vaccination in rural Sindh, Pakistan: A case of refusal, ignorance or access?. Vaccine.

[21] Riaz A., Husain S., Yousafzai M.T., Nisar I., Shaheen F., Mahesar W. (2018). Reasons for non-vaccination and incomplete vaccinations among children in Pakistan. Vaccine.

[22] Vikram K., Vanneman R., Desai S. (2012). Linkages between maternal education and childhood immunization in India. Soc Sci Med.

[23] Bawa S., Shuaib F., Saidu M., Ningi A., Abdullahi S., Abba B. (2018). Conduct of vaccination in hard-to-reach areas to address potential polio reservoir areas, 2014–2015. BMC Public Health.

[24] Kazi A.M., Ahsan N., Jamal S., Khan A., Mughis W., Allana R. (2021). Characteristics of mobile phone access and usage among caregivers in Pakistan – A mHealth survey of urban and rural population. Int J Med Inf.

[25] Kazi A.M., Ali M., Zubair K., Kalimuddin H., Kazi A.N., Iqbal S.P. (2018). Effect of mobile phone text message reminders on routine immunization uptake in Pakistan: Randomized controlled trial. JMIR Public Health Surveill.

[26] Chandir S., Siddiqi D.A., Abdullah S., Duflo E., Khan A.J., Glennerster R. (2022). Small mobile conditional cash transfers (mCCTs) of different amounts, schedules and design to improve routine childhood immunization coverage and timeliness of children aged 0–23 months in Pakistan: An open label multi-arm randomized controlled trial. EClinicalMedicine.

[27] van der Kroon B., Brouwer R., van Beukering P.J.H. (2013). The energy ladder: Theoretical myth or empirical truth? Results from a meta-analysis. Renew Sustain Energy Rev.

[28] Over 20 million children worldwide missed out on measles vaccine annually in past 8 years, creating a pathway to current global outbreaks. 2019 [press release].

[29] Hussain I., Khan A., Rhoda D.A., Ahmed I., Umer M., Ansari U. (2023). Routine immunization coverage and immunization card retention in Pakistan: Results from a cross-sectional national survey. Pediatr Infect Dis J.

[30] Roy P., Vekemans J., Clark A., Sanderson C., Harris R.C., White R.G. (2019). Potential effect of age of BCG vaccination on global paediatric tuberculosis mortality: A modelling study. Lancet Glob Health.

[31] Kurosky S.K., Davis K.L., Krishnarajah G. (2017). Effect of combination vaccines on completion and compliance of childhood vaccinations in the United States. Hum Vaccin Immunother.

[32] Kalies H., Grote V., Verstraeten T., Hessel L., Schmitt H.-J., von Kries R. (2006). The use of combination vaccines has improved timeliness of vaccination in children. Pediatr Infect Dis J.

[33] Sheikh S.S., Ali S.A. (2014). Predictors of vaccination card retention in children 12–59 months old in Karachi. Pakistan Oman Med J.

