# Supplementary material for: Differential coverage for vaccines in the expanded program on immunization (EPI) among children in rural Pakistan
Source: Vaccine. 2023 Apr 17;41(16):2680–9. doi: 10.1016/j.vaccine.2023.03.007 (PMC10124121; doi:10.1016/j.vaccine.2023.03.007)
Supplement: Supplementary data 1 [file mmc1.docx]

**Supplementary table S1**
